# Rational Use of Monoclonal Antibodies as Therapeutic Treatment in an Oncologic Patient with Long COVID

**DOI:** 10.3390/v15030614

**Published:** 2023-02-23

**Authors:** Maria Grazia Cusi, Anna Maria Di Giacomo, Gabriele Anichini, Gianni Gori Savellini, Chiara Terrosi, Claudia Gandolfo, Michele Maio

**Affiliations:** 1 Virology Unit, Department of Medical Biotechnologies, University of Siena, 53100 Siena, Italy; 2 Virology Unit, Department of Medical Biotechnolopgies, Santa Maria Alle Scotte University Hospital, V.le Bracci 16, 53100 Siena, Italy; 3 Center for Immuno-Oncology, Medical Oncology and Immunotherapy, Department of Medical Sciences, Surgical and Neuroscience, 53100 Siena, Italy; 4 Italian Network for Tumor Bio-Immunotherapy Foundation Onlus, 53100 Siena, Italy

**Keywords:** long COVID, monoclonal antibodies, resistance, post-exposure treatment

## Abstract

We present the case of a 76-year-old male patient persistently infected by severe acute respiratory syndrome coronavirus 2 (SARS-CoV-2) in the setting of a stage IIIC cutaneous melanoma and non-Hodgkin’s lymphoma (NHL). Due to the persistent coronavirus disease 19 (COVID-19), all cancer treatments were discontinued. Because of the worsening of his clinical state and the persistence of SARS-CoV-2 positivity for more than six months, the patient was treated with sotrovimab, which was ineffective due to resistance mutations acquired during that time. In order to resume cancer treatment and make the patient free from SARS-CoV-2, an in vitro screening of Evusheld monoclonal antibodies (tixagevumab–cilgavimab) against the viral strains isolated from the subject was performed. The promising results obtained during in vitro testing led to the authorization of the off-label use of Evusheld, which made the patient negative for SARS-CoV-2, thus, allowing him to resume his cancer treatment. This study highlights the Evusheld monoclonal antibodies’ efficacy, not only in prevention but also in successful therapy against prolonged COVID-19. Therefore, testing neutralizing monoclonal antibodies in vitro against SARS-CoV-2 mutants directly isolated from patients could provide useful information for the treatment of people affected by long COVID.

## 1. Introduction:

Since the beginning of the SARS-CoV-2 pandemic, many vaccines have been developed in record time to contrast the spreading of the virus and, overall, to prevent serious disease or even death in humans [1]. However, immunocompromised people who are subjected to a treatment that can blunt the humoral response to vaccines are at major risk of developing the disease and facing serious, life-threatening complications. Among them, patients with cancer are at higher risk, and those treated with immunosuppressors or B-cell depleters may show a low humoral response after being vaccinated against SARS-CoV-2 [2]. In this regard, substantial help is provided by monoclonal antibodies, directed primarily against the receptor binding domain (RBD) of the SARS-CoV-2 Spike glycoprotein, thus inhibiting the interaction between RBD and the ACE-2 receptor and neutralizing the ability of the virus to bind and fuse with the host target cells. Thus, monoclonal antibodies are able to provide rapid protection in an emergency either for pre- (tixagevumab–cilgavimab, Evusheld) and post-exposure (REGEN-COV) prophylaxis against COVID-19, although many of them have lost their ability to neutralize the Omicron variants [3,4,5] because of the advent of numerous sublineages with critical aminoacid mutations in the Spike receptor-binding domain (RBD). Among them, tixagevimab–cilgavimab (Evusheld, AZD7442) is a long-acting monoclonal antibody combination, which has been licensed as pre-exposure prophylaxis to prevent COVID-19 in people with moderate to severe immune impairment. When this study was conducted, Evusheld was the only option for the pre-exposure prophylaxis of COVID-19 [6].

## 2. Methods

### 2.1. Patient History

The subject examined in this case report is a 76-year-old male patient who was diagnosed with stage IIIc cutaneous melanoma in July 2006. On November 2016, a follow-up CT scan showed a recurrence on the right axillary and abdominal lymph nodes and kidney. He was enrolled in a phase III trial and received nivolumab (1 mg) combined with Ipilimumab (3 mg) every 3 weeks for 4 doses; then, he received nivolumab (480 mg) every 4 weeks until October 2018, achieving a stable disease (SD) (defined according to RECIST v.1.1 criteria).

On November 2018, due to a confirmed progression of disease (PD) (defined according to RECIST v.1.1 criteria), he was treated with i.v. ipilimumab (3 mg) every 3 weeks for 4 cycles until March 2019, achieving SD. Due to latero-cervical lymph node PD, on November 2019, he underwent radiotherapy on thw right latero-cervical lymph nodes, achieving a partial response.

On February 2020, due to a lymph nodal PD, the patient was enrolled in a phase I IOA-244-101 trial, starting on IOA-244, an orally bioavailable, selective PI3Kδ inhibitor, achieving a durable, stable disease (13 months). In October 2021, in light of a concurrent diagnosis of non-Hodgkin lymphoma (NHL) by axillary lymph node biopsy, treatment with rituximab 375 mg/mq was started, with evidence of SD.

On 17 December 2021, he was SARS-CoV-2 positive by molecular test. For convenience, this date was defined as Day 0 of infection.

Then, due to the persistent COVID infection, all cancer treatments were discontinued. All nasopharyngeal swabs collected from the subject were analysed for the presence of the SARS-CoV-2 genome using the Xpert Xpress SARS-CoV-2 test, an RT-PCR test targeting the E and N2 proteins of the virus. Samples were run on a GeneXpert Dx system (Cepheid, Sunnyvale, CA, USA).

This research was carried out according to the principles of the Helsinki declaration, with reference to the BIOBANK MIU-2010 document approved by the Ethics Committee with amendment No. 1 on 17 February 2020. Prior to participating in this study, the subject signed a written informed consent.

### 2.2. SARS-CoV-2 IgG Antibody Detection

In order to evaluate the humoral response against SARS-CoV-2 in our patient, whole blood samples were collected and centrifuged at 1600× *g* for 15 min to separate the serum. Then, subject sera were analyzed using the Abbott SARS-CoV-2 IgGII Quant assay (Abbott Laboratories, Chicago, IL, USA), a chemiluminescent microparticle immunoassay (CMIA), in order to evaluate the immune status with quantitative measurement of IgG antibodies against the spike receptor-binding domain (RBD) of SARS-CoV-2. This assay, routinely used in our laboratory for the evaluation of the immune status of infected and vaccinated individuals, was performed on an Abbott Alinity (Abbott Diagnostics) instrument according to the manufacturer’s instructions. A sample was considered positive when the result was >50.0 AU/mL, with a limit of detection of 40,000 AU/mL.

### 2.3. SARS-CoV-2 Microneutralization Test

A SARS-CoV-2 virus neutralization assay was carried out on Vero E6 cells in a 96-well microplate. Briefly, twenty-five microliters of two-fold serial dilutions (1:8 to 1:1024) of sera samples (drawn post-sotrovimab, 14 and 30 days post-Evusheld treatment) were added to an equal volume of SARS-CoV-2 B.1.617.2 (SARS-CoV-2/human/ITA/TUS-Siena-40/2021; GenBank: OM736177.1), Isolate 1 (SARS-CoV-2/human/ITA/TUS-Siena5395733/2022, GenBank: OP583725.1), Isolate 2 (SARS-CoV-2/human/ITA/TUS-Siena5417371/2022, GenBank: OP583740.1), and Isolate 3 (SARS-CoV-2/human/ITA/TUS-Siena5448809/2022, GenBank: OP583739.1) containing 100 TCID_50_ and incubated for 90 min at 37 °C. Finally, 50 μL of Vero E6 cell suspension (2 × 10^5^ cells/mL) prepared in complete DMEM was added to each well. After incubation at 37 °C, cultures were examined daily for the presence of CPE under a microscope (Olympus IX51). The 50% endpoint titer was calculated by using the Reed–Muench method [7]. Positive and negative control sera were included in each assay [8,9]. Geometric mean titers (GMTs) of the neutralization assays were calculated. Each result was reported as the average of three replicates.

### 2.4. SARS-CoV-2 Whole Genome Sequencing

For the sequencing of SARS-CoV-2 isolates, nasopharyngeal swabs were collected from the patient on 17 January (Day 37, SARS-CoV-2/human/ITA/TUS-Siena5322931/2022, GenBank: OP581934.1), 4 April (Day 114, SARS-CoV-2/human/ITA/TUS-Siena5382804/2022, GenBank: OP583726.1), 21 April (Day 131, Isolate 1, SARS-CoV-2/human/ITA/TUS-Siena5395733/2022, GenBank: OP583725.1), 30 May (Day 170, Isolate 2, SARS-CoV-2/human/ITA/TUS-Siena5417371/2022, GenBank: OP583740.1), and 25 July 2022 (Day 226, Isolate 3, SARS-CoV-2/human/ITA/TUS-Siena5448809/2022, GenBank: OP583739.1). Total RNA was isolated from the patient swab using the RNeasy Mini Kit (Qiagen Inc., Hilden, Germany) according to the manufacturer’s instructions. Then, ten μL of total RNA were retrotranscribed using FastGene Scriptase II (Nippon Genetics EUROPE, Düren, Germany). Whole-genome sequencing (WGS) of SARS-CoV-2 was performed with the EasySeq RC-PCR SARS-CoV-2 (novel coronavirus) Whole-Genome Sequencing kit (NimaGen, Netherlands), enabling the amplification of overlapping fragments around 300 bp to cover the entire viral genome. Libraries were quantified using Qubit dsDNA HS (High Sensitivity) Assay Kit on a Qubit fluorometer (Invitrogen, Waltham, MA, USA). Finally, 10 pM libraries, added with 3% of PhiX v3 control (Illumina, San Diego, CA, USA), were sequenced on an Illumina MiSeq platform with a 2 × 150 bp paired-end protocol using a MiSeq Reagent Nano Kit v2 (300-cycles) (Illumina, USA). Raw data were collected and analysed using a specific SARS-CoV-2 workflow on virSEAK 0.12.1 software (JSI medical systems GmbH, Ettenheim, Germany). After quality check and adapter trimming, the sequences were mapped to the SARS-CoV-2 reference MN908947.3 with the identification of the nucleotide variants and amino acid changes and relative coverage. Finally, all the sequences were deposited both in the GenBank (ID reported) and GISAID databases.

## 3. Results

In this study, we analysed and monitored a 76-year-old male patient who was diagnosed with stage IIIC cutaneous melanoma in July 2006. Due to a nodal and renal recurrence, he received three lines of treatment beginning in November 2016, achieving multiple and durable stabilization of the disease. From October 2021, in light of a concurrent diagnosis of non-Hodgkin’s lymphoma (NHL) by axillary lymph node biopsy, treatment with rituximab 375 mg/mq was started, with evidence of stable disease (according to RECIST v.1.1 criteria).

Despite receiving the third dose of the vaccine on 29 September 2021, the patient was negative for the presence of specific antibodies (anti-Spike IgG CMIA 1.7 AU/mL) to the Wuhan reference and did not show neutralizing antibodies against the Wuhan, Delta, and Omicron BA.1 strains after two months. In December 2021, after being infected with the SARS-CoV-2 Delta (AY.4.2) variant, all cancer treatments were discontinued. Due to the persistence of viral infection, the SARS-CoV-2 virus was isolated from the patient’s nasopharyngeal swab and sequenced in the samples drawn on Day 37, Day 114, Day 131 (Isolate 1), Day 170 (Isolate 2), and Day 226 (Isolate 3) (Table 1). Sequencing revealed the presence of further mutations accumulated in the Spike sequence (Table 1).

Because of the worsening of his clinical state and the persistence of SARS-CoV-2 positivity, the patient was suggested to be treated with sotrovimab, a monoclonal antibody that neutralizes many sarbecoviruses, including SARS-CoV-2 and the Delta variant, by binding to a highly conserved epitope within the receptor-binding domain (RBD) [10].

The patient was treated with 500 mg as a single IV infusion on 2 May 2022. He continued being positive for SARS-CoV-2 in the samples drawn in the following three weeks. Thus, the virus was isolated from the swab in vitro and sequenced (Isolate 2). We found peculiar amino acid mutations in the Spike domain related to the use of Sotrovimab, as reported in the literature [11,12]. Indeed, the mutation E340A in the Spike domain, associated with a strong reduction in neutralization by sotrovimab, appeared after treatment (Table 1). By contrast, some mutations in the RBD (receptor-binding domain), corresponding to E484G, together with deletions S241–243, already present in the sample drawn on Day 114, could be responsible for the immune evasion, as previously reported [13,14]. Later on, Isolate 3 presented the T478I mutation within the RBD, with 32% of the viral population having a switch to isoleucine, which, being a nonpolar amino acid similar to leucine, conferred to the Delta variant resistance to sotrovimab [15].

In order to restart cancer treatment in this patient and make him recover from SARS-CoV-2, we tested three of his viral isolates (no.1, pre-sotrovimab; no.2, post-sotrovimab and no.3, pre-Evusheld) in vitro (Table 2), in addition to the original Delta variant (B.1.617.2), with a hyperimmune serum of a subject previously infected with the Delta variant (neutralizing GMT = 1/512), with sotrovimab (using a starting point concentration of 300 ng/mL) and Evusheld (tixagevumab–cilgavimab, using a starting point concentration of 600 ng/mL in total) (Table 3) [16]. The human serum inhibited the original Delta variant, as well as the other two isolates of the patient, with a neutralizing GMT of 1/362 (*p* < 0.05), but it reacted at a lesser extent with Isolate 3. Sotrovimab, which reached an effective concentration (EC_50_ with 37.5 ng/mL) against the Delta variant, was not able to neutralize the two patient’s isolates (GMT < 1/8), indicating that the mutations acquired later could probably be involved in immune escape. On the contrary, Evusheld was still able to neutralize the three virus isolates at a concentration of 9.3 ng/mL.

These promising results induced us to request an off-label use of Evusheld (300 mg/300 mg) for this patient since such a monoclonal antibody combination is recommended only to prevent SARS-CoV-2 disease progression and has an extended half-life of approximately 90 days in humans [17]. The patient became negative for SARS-CoV-2 by molecular testing one week after treatment. A month later, still being negative, he was able to start cancer treatment again. He continued to test negative three months after this treatment.

## 4. Discussion

Recent studies have suggested that up to 20% of overall COVID-19 patients develop prolonged COVID-19 [18]. In the context of cancer, oncologic patients have been found to be more susceptible to COVID-19 infection [19]. Indeed, about 13% of patients receiving systemic anti-cancer therapy had to stop their treatment permanently when they were diagnosed with COVID-19. Nearly 16% of them had to adjust their cancer treatment due to COVID-19 [18]. Worryingly, the decrease in treatment of cancers seen during the COVID-19 pandemic potentially lead to a surge in cancer progression. The impacts of delays, interruptions, and cancellations of cancer care services at the initial stages of the pandemic have been observed by oncological centers worldwide. Fortunately, owing to a concerted global effort, several highly effective vaccines have been developed at an unprecedented speed [20,21]. In large parts of the world, mass vaccination campaigns have considerably reduced the incidence of severe COVID-19 in the general population after at least two vaccine doses [22]. Owing to the high risk of developing severe COVID-19, patients with cancer were prioritized for vaccination in most countries [23]. However, the antibody response of immunized cancer patients was often delayed and diminished, mainly in patients receiving chemotherapy or rituximab, resulting in breakthrough infections. Observational data suggest that serological responses to vaccines may be blunted in patients who are immunocompromised [24]. However, vaccination is still recommended for these patients because it may provide partial protection, including cell-mediated immune protection. Indeed, the patient analysed in this study was protected by the vaccine, which was successful in the prevention of severe illness. The patient was not treated with paxlovid because it should be administered as soon as possible after a diagnosis of COVID-19 and within 5 days of symptom onset. In this case, it was no longer possible to treat the patient because of a late diagnosis of SARS-CoV-2 infection. Paxlovid, consisting of nirmatrelvir and low-dose ritonavir, inhibits the viral replication of SARS-CoV-2 by blocking the activity of the SARS-CoV-2-3CL protease. Coadministration with low-dose ritonavir slows the metabolism of nirmatrelvir and prolongs its activity [25]. Therefore, the other option for the treatment of the patient with COVID-19 was the use of monoclonal antibodies. Sotrovimab, a recombinant human monoclonal antibody (mAb) against SARS-CoV-2, had US FDA Emergency Use Authorization (EUA) for the treatment of high-risk outpatients with mild-to-moderate COVID-19 treatment. Such a monoclonal antibody was associated with a reduced risk of hospitalization and mortality compared with no mAb treatment, and its clinical effectiveness persisted throughout the months [26]. Since Sotrovimab was very efficacious against the Delta variant [27], the patient was treated with this monoclonal antibody. Unfortunately, the patient did not respond to the treatment and developed Spike gene mutations associated with high-level sotrovimab resistance in vitro. These findings underscore the importance of the stewardship of monoclonal antibodies, particularly because sotrovimab is one of the few monoclonal antibodies with retained activity against the BA.1 variant [12].

For this reason, a combination of two monoclonal antibodies, tixagevimab and cilgavimab (Evusheld), that bind the spike protein and prevent the virus from entering human cells, was tested off-label since it is recommended for preventing COVID-19 in adults and adolescents who do not require supplemental oxygen and who have an increased risk of developing severe disease.

Unlike vaccines, Evusheld does not depend on a healthy immune system to generate protective immunity, and although Evusheld is known to be effective against different variants, it is not yet known how long this protection lasts. Our results showed that Evusheld can be efficacious not only in prevention but also in successful therapy, as described in this case report. The possibility of testing available neutralizing monoclonal antibodies in vitro against SARS-CoV-2 mutants directly isolated from patients, as was done in this study, could provide useful information for the treatment of people affected by long COVID. Moreover, it is worth noting that the availability of different monoclonal antibodies against SARS-CoV-2 allows for testing them with single variants isolated from the patient, as mAbs evidently represent strong weapons against COVID-19. This approach is extremely useful when people are frail or subjected to oncologic or immunosuppressive therapy. This report concerns only a single patient; therefore, larger double-blind, placebo-controlled trials of using Evusheld or other combinations of monoclonal antibodies as post-exposure treatment in patients with long COVID-19 are necessary to draw formal conclusions.

## Figures and Tables

**Table 1:** Acquired mutations in the Spike protein observed during patient monitoring, with relative prevalence (%). All the five strains containing the listed mutations in the Spike gene belong to the AY.4.2 (B.1.617.2.4.2) strain, with 99.9% similarity throughout the whole genome compared to the original viral sequence (Day 37), with a maximum of 20 amino acid mutations.

	Day 37	Day 114	Day 131 (Isolate 1)	Day 170 (Isolate 2)	Day 226 (Isolate 3)
Spike: P25L					100%
Spike: T95I	100%	100%	100%	100%	
Spike: T95S					100%
Spike: Y144H				100%	100%
Spike: del 241–243		100%	100%	100%	100%
Spike: E340A				25%	100%
Spike: T478K	100%	100%	100%	100%	
Spike: T478I					I 32%
Spike: N501Y					100%
Spike: E484G		100%	100%	100%	
Spike: Q613H				100%	100%
Spike: P681R					100%
Spike: A684V					100%

Pre-existing Spike mutations: T19R, G142D, Y145H, E156G, del 157–158, A222V, L452R, D614G, D950N, V1164L.

**Table 2:** Neutralizing antibody titers of patient sera tested either against Delta (B.1.617.2) strain and the three different patient isolates (pre- and post-sotrovimab, pre-Evusheld). All experiments were performed in triplicate. Statistical differences among GMTs were evaluated with respect to Isolate 1 (* *p* < 0.05; ** *p* < 0.01; *** *p* < 0.001).

	Neutralizing Antibody Titer (GMT) vs. Viral Isolates			
	Human Polyclonal Serum	Patient Sera		
Virus isolate	Delta (B.1.617.2) (IgG 5900.7 AU/mL)	Post Sotrovimab (IgG 4814.5 AU/mL)	14 days post Evusheld (IgG 32,241.0 AU/mL)	30 days post Evusheld (IgG 21,513.4 AU/mL)
Delta (B.1.617.2)	1/512	/	/	/
Isolate 1 Pre-Sotrovimab	1/362	1/45	1/512	1/203
Isolate 2 Post-Sotrovimab	1/362	<1/8 ***	1/362 *	1/181
Isolate 3 Pre-Evusheld	1/23 ***	<1/8 ***	1/161 ***	1/102 **

**Table 3:** Half-maximal effective concentration (EC_50_) of tested monoclonal antibodies (sotrovimab, Evusheld) against the three different patient isolates (pre- and post-sotrovimab, pre-Evusheld) and the Delta strain (B.1.617.2). Sotrovimab concentration range: 300–2.1 ng/µL; Evusheld: 600 (300 + 300 tixagevumab–cilgavimab)-4.15 (2.07 + 2.07) ng/µL [16].

	Monoclonal Antibody Tested	
Virus Isolate	Sotrovimab (EC_50_, ng/µL)	Evusheld (EC_50_, ng/µL)
Delta (B.1.617.2)	37.5	9.3
Isolate 1 Pre-Sotrovimab	>300	9.3
Isolate 2 Post-Sotrovimab	>300	9.3
Isolate 3 Pre-Evusheld	>300	9.3

## Data Availability

Data supporting reported results can be provided, upon reasonable request, by contacting the following address (mariagrazia.cusi@unisi.it).
